# Divergent Cellular Expression Patterns of PD-L1 and PD-L2 Proteins in Breast Cancer

**DOI:** 10.3390/jpm14050478

**Published:** 2024-04-29

**Authors:** Julie M. Jorns, Yunguang Sun, Sailaja Kamaraju, Yee Chung Cheng, Amanda Kong, Tina Yen, Caitlin R. Patten, Chandler S. Cortina, Christopher R. Chitambar, Hallgeir Rui, Lubna N. Chaudhary

**Affiliations:** 1 Department of Pathology, Froedtert and Medical College of Wisconsin, Milwaukee, WI 53226, USA; ysun@mcw.edu; 2 Division of Hematology and Oncology, Department of Medicine, Froedtert and Medical College of Wisconsin, Milwaukee, WI 53226, USA; skamaraju@mcw.edu (S.K.); ycheng@mcw.edu (Y.C.C.); cchitamb@mcw.edu (C.R.C.); lchaudhary@mcw.edu (L.N.C.); 3 Division of Surgical Oncology, Department of Surgery, Froedtert and Medical College of Wisconsin, Milwaukee, WI 53226, USA; akong@mcw.edu (A.K.); tyen@mcw.edu (T.Y.); ccortina@mcw.edu (C.S.C.); 4 Department of Pharmacology, Physiology & Cancer Biology, Sidney Kimmel Medical College, Thomas Jefferson University, Philadelphia, PA 19144, USA; hallgeir.rui@jefferson.edu; 5 Sidney Kimmel Cancer Center, Philadelphia, PA 19144, USA

**Keywords:** PD-L1, PD-L2, breast cancer, immunohistochemistry, immune checkpoint inhibition

## Abstract

PD-L1 immunohistochemistry (IHC) has become an established method for predicting cancer response to targeted anti-PD1 immunotherapies, including breast cancer (BC). The alternative PD-1 ligand, PD-L2, remains understudied but may be a complementary predictive marker. Prospective analysis of 32 breast cancers revealed divergent expression patterns of PD-L1 and PD-L2. PD-L1-positivity was higher in immune cells than in cancer cells (median = 5.0% vs. 0.0%; *p* = 0.001), whereas PD-L2-positivity was higher in cancer cells than immune cells (median = 30% vs. 5.0%; *p* = 0.001). Percent positivity of PD-L1 and PD-L2 were not correlated, neither in cancer cells nor immune cells. Based on a cut-point of ≥1% positivity, ER+ tumors (n = 23) were frequently PD-L2-positive (73.9%), whereas only 40.9% were PD-L1-positive. These data suggest differential control of cellular PD-L1 and PD-L2 expression in BC and a potential role for PD-L2 IHC as a complementary marker to PD-L1 to improve selection of aggressive ER+ BC that may benefit from anti-PD-1 therapy.

## 1. Introduction

The discovery of cancer progression mechanisms of immune evasion via upregulation of programmed cell death-1 (PD-1) ligand-1 (PD-L1) has led to targeted immunotherapies blocking the PD-1 axis [1]. For breast cancer (BC), KEYNOTE-086 and KEYNOTE-522 clinical trials led to FDA approval of pembrolizumab, a PD-1 inhibitor (PD-1i), in the treatment of patients with triple-negative breast cancer (TNBC). While PD-L1 expression in cancer cells and/or cancer-associated stromal immune cells has been associated with the therapeutic response to PD-1i in many settings, PD-L1-positivity is only moderately predictive of response in BC. In KEYNOTE-355, patients with metastatic TNBC receiving pembrolizumab showed improved progression-free survival (PFS), with greater benefit in PD-L1-positive patients [2]. However, KEYNOTE-522 patients with TNBC treated with neoadjuvant chemotherapy (NACT) with pembrolizumab showed a higher rate of pathologic complete response, regardless of PD-L1 status [3]. Therefore, most early-stage patients with TNBC are currently offered NACT/PD-1i regardless of PD-L1 tumor status.

Durable responses to anti-PD1 therapies have been observed but are less common in estrogen-receptor-positive (ER+) BC compared to TNBC [4]. We recently reported that high expression of PD-L2 in cancer cells of treatment-naïve ER+ BC was an independent predictor of shorter PFS [5]. Importantly, PD-L2 is an alternative but understudied PD-1 ligand that has an approximately three-fold higher affinity for PD-1 than PD-L1 [6]. PD-L2, therefore, likely has key roles in BC, and combined PD-L1/PD-L2 status may help improve selection for PD-1i therapy. Therefore, we aimed to determine baseline expression patterns of PD-L1 and PD-L2 proteins in BC.

## 2. Materials and Methods

Diagnostic core biopsies from 31 consecutive treatment-naïve patients diagnosed with localized or locoregional ER+/HER2- BC or TNBC, being screened for our ongoing study (NCT04243616), were prospectively analyzed for PD-L1 and PD-L2 protein expression by immunohistochemistry (IHC) using validated antibodies (PD-L1—rabbit monoclonal antibody, 73-10, RTU, Leica, Deer Park, IL; PD-L2—rabbit polyclonal antibody, Sigma-Cat#SAB3500395, 1:200 dilution [5]). Percent positivity of PD-L1 and PD-L2 in cancer cells and immune cells was visually determined by a board-certified, fellowship-trained breast pathologist (J.M.J.). Whole slide sections were utilized, and the entire tumor biopsy region was assessed. On-slide tonsil and placenta were used as positive controls for PD-L1 and PD-L2, respectively. Tumor status was considered positive if detectable membranous PD-L1 or membranous and/or cytoplasmic PD-L2 expression was present in ≥1% of cancer cells or stromal immune cells. Cellular positivity was further quantified as 0%, <1%, 1%, 5%, or 10%, and then by 10% increments. Non-negligible scores of <1% were assigned random numbers >0%<1% for statistical purposes. ER and HER2 status of tumors was defined per current CAP/ASCO guidelines. A pre-specified sample size calculation for power analysis was not performed for this discordance analysis, as there was no available pilot data to base a statistical power calculation on. Spearman rank analysis was used to test correlation between PD-L1 and PD-L2 protein expression levels in cancer cells and immune cells, and differences in the levels of PD-L1 and PD-L2 were compared by the Wilcoxon rank sum test.

## 3. Results

**Figure 1:**
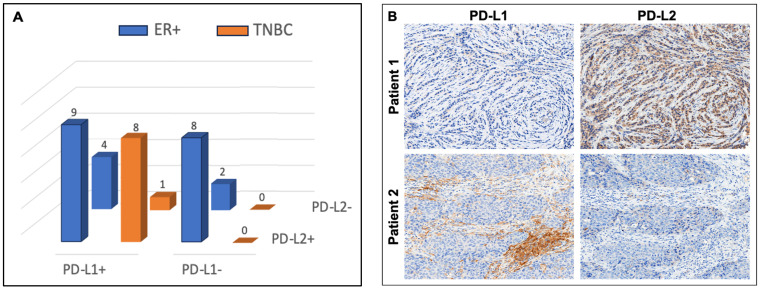
(**A**) PD-L1 and PD-L2 status by breast cancer biomarker subtype. Estrogen-receptor-positive (ER+) and triple-negative breast cancer (TNBC) (N = 32). (**B**) Examples of discordant expression patterns of PD-L1 and PD-L2 in breast cancer. Patient 1 had ER-positive, HER2-negative invasive lobular carcinoma, which showed low PD-L1 and high PD-L2, predominantly localized to cancer cells. Patient 2 had ER-positive, HER2-negative invasive ductal carcinoma, which showed high PD-L1, predominantly localized to stromal immune cells, and low PD-L2.

PD-L1 and PD-L2 protein expression was analyzed in 32 tumors from 31 female patients, including 23 (71.9%) ER+ BC and 9 (28.1%) TNBC, with 1 patient having multifocal unilateral ER+ BC. Demographic and pathologic features are included in Table 1. By applying the conventional threshold for tumor PD-L1-positivity of ≥1% in cancer cells or stromal immune cells, we found that all nine TNBC tumors were PD-L1-positive, with eight (88.9%) of these also being PD-L2-positive. Of the 23 ER+ tumors, 17 (73.9%) were PD-L2-positive, of which only 9 (39.1%) were also PD-L1-positive. Among the 10 PD-L1-negative tumors, 8 (80.0%) were PD-L2-positive, all of which were also ER+ (Figure 1A).

**Figure 2:**
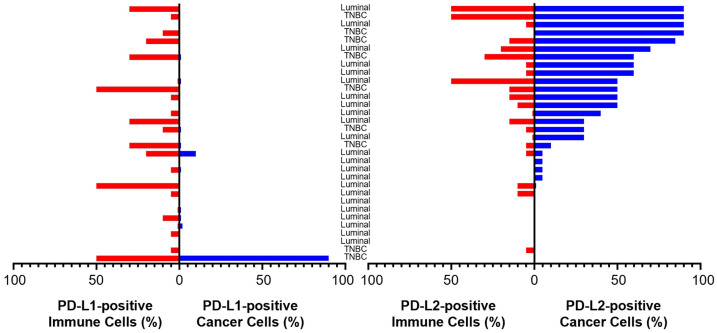
Expression of PD-L1 and PD-L2 in immune cells and cancer cells of a prospective series of malignant breast tumors. Tumors were scored for percent positivity for PD-L1 (left panel) and PD-L2 (right panel) in stromal immune cells (red bars) and cancer cells (blue bars). Tumors are denoted as ER+ or TNBC and ordered by increasing cancer cell levels of PD-L2 for ease of interpretation.

Cellular expression patterns of PD-L1 and PD-L2 proteins in malignant breast tumors were distinctly different (Figure 1B). While PD-L1 was predominantly expressed in stromal immune cells, PD-L2 was predominantly expressed in cancer cells. When analyzed across all 32 tumors, the percentage of PD-L1-positive immune cells was higher than the percentage of PD-L1-positive cancer cells (median = 5.0% vs. 0.0%; *p* = 0.001). In contrast, percent PD-L2-positivity was higher in cancer cells than in immune cells (median = 30% vs. 5.0%; *p* < 0.001; Figure 2). Among PD-L1-negative ER+ tumors, most displayed marked cancer cell positivity for PD-L2 (≥30% in 6 of 8).

Overall, percent PD-L1-positivity in immune cells or cancer cells did not correlate with percent PD-L2-positivity in either cell type. Percent positivity for PD-L2 in immune cells and cancer cells was strongly correlated (rho = 0.61, *p* < 0.001), whereas the corresponding PD-L1 values were not. By tumor type, PD-L1 positivity in cancer cells and immune cells was positively correlated (rho = 0.69, *p* = 0.04) in ER+ but not TNBC. Conversely, within ER+ BC, but not within TNBC, PD-L2 positivity in cancer cells and immune cells was positively correlated (rho = 0.68, *p* < 0.001). PD-L1 displayed lower positivity in immune cells in ER+ BC than TNBC (median = 1% vs. 20%; *p* = 0.011), whereas the PD-L2-positivity in cancer cells or immune cells did not differ significantly between ER+ BC and TNBC.

## 4. Discussion

In this study, PD-L1 and PD-L2 proteins showed divergent expression in BC, as evidenced by a lack of significant correlation and by striking differences in cellular expression patterns. While PD-L1 expression was mainly observed in stromal immune cells in BC, we detected PD-L2 expression predominantly in cancer cells. TNBC was generally positive for both PD-L1 and PD-L2, whereas one-third (8 of 23) of ER+ BC were positive for PD-L2 but negative for PD-L1. While the ≥1% threshold used for positive tumor status for PD-L1 or PD-L2 is lenient, the identification of PD-L1-negative ER+ tumors that expressed significant levels of PD-L2-positive cancer cells supported the possibility that combined analysis of PD-L1 and PD-L2 will better predict the response to PD-1i than PD-L1 alone, and may be of value in determining treatment options for aggressive ER+ tumors. PD-L2 status of tumors may also be informative of the efficacy of agents that target PD-L1 alone, e.g., atezolizumab. However, studies are needed to validate how effectively combined PD-L1/PD-L2 analysis will predict the response to anti-PD-1 therapies.

Studies of PD-L1 have overshadowed the literature to date on PD-L2 in BC and other tumors. A report on 192 patients found detectable PD-L2 in 50.8% of tumors by pathologist evaluation of chromogen IHC, without association of tumor PD-L2 scores with clinical outcomes [7]. However, this cohort included all BC subtypes. Another study of 177 patients treated with NACT without targeted anti-PD1 therapy, 38.4% of which were ER+ BC, also reported no association between PD-L2 and clinical outcome; however, in that study, ER+ patients were grouped with HER2+ for response assessment in “non-TNBC” versus TNBC, which might have impacted the data interpretation [8]. In contrast, we recently determined that elevated expression of PD-L2 protein in cancer cells of one-third of ER+ BC, as identified by quantitative fluorescence IHC, was an independent marker of unfavorable prognosis, as validated in independent cohorts from two different institutions based on analyses of 954 patients [5]. Notably, one-third of the current cohort of ER+ tumors with the highest tumor PD-L2 levels displayed significant cancer cell PD-L2-positivity of ≥50%. 

PD-L2 and other immune checkpoint proteins may partly explain why PD-L1 IHC has been only modestly predictive of PD-1i therapy outcomes in BC and other malignancies. However, PD-L1 assessment has also been challenged by evolving recommendations and practices. Companion diagnostic assays developed in parallel to anti-PD-1 antibodies require a particular platform and antibody for testing. Labs must then validate multiple PD-L1 assays to assess PD-L1 expression in various tumors, which is often not practical. Fortunately, studies on BC have shown relative concordance among assays, ranging from good to excellent [9,10]. However, pathologist interpretation is also subject to inter- and intra-observer variability, with concordance ranging from poor to excellent, depending on the setting [11,12]. More significant discordance and worse reproducibility were reported among low-expressing cases with PD-L1 around the cutoff of 1% [12], which is made more challenging when attempting to assess whether immune cells are in close enough proximity to the cancer cells to warrant inclusion in scoring. PD-L2 assessment may show similar susceptibility to interpretative differences, but a predictive scoring cutoff may prove less problematic to establish due to higher PD-L2 staining in cancer cells than in immune cells. 

This study is limited by a relatively small sample size and regional assessment at one academic and two community hospitals within our healthcare system. Analytic and interpretive variability was minimized by prospective evaluation of biopsy specimens obtained and processed in a standard fashion with nominal cold ischemia time, and IHC assessment by an experienced breast pathologist. Notably, we used the PD-L1 clone 73-10, which has not been approved for BC but has been used in the assessment of lung cancer and is comparable to the Dako/Agilent 22C3 assay [13], an approved companion diagnostic for pembrolizumab in the treatment of TNBC. 

## 5. Conclusions

In summary, our finding of frequent PD-L1/PD-L2 discordance in BC supports the potential value of PD-L2 as a complementary marker when evaluating breast tumors for immune checkpoint inhibitor therapy. PD-L2 IHC may particularly benefit BC patients who are eligible for chemotherapy with aggressive ER+ tumors positive for PD-L2 protein. Retrospective analysis of PD-L2 in the tumors from the Phase III KEYNOTE-756 [14] and Checkmate-7FL [15] clinical trials may help explain the improved rates of pathological complete response to PD-1i in ER+ BC.

## Figures and Tables

**Table 1:** Clinicopathologic features of clinical trial patients (N = 32 tumors from 31 patients).

Feature	Value
Age (years) (median, range)	49.4 (26.2–73.4)
Race (N, %)	
White	25 (80.7)
Black	5 (16.1)
Other (unspecified)	1 (3.2)
cT Stage (N, %)	
cTx	1 (3.2)
cT1	5 (16.1)
cT2	13 (42)
cT3	12 (38.7)
cN Stage (N, %)	
cN0	10 (32.3)
cN1	19 (61.3)
cN2	1 (3.2)
cN3	1 (3.2)
Histology (N, %)	
Invasive Ductal Carcinoma	27 (84.4)
Invasive Lobular Carcinoma	5 (15.6)
Nottingham Grade (N, %)	
1 (Well Differentiated)	4 (12.5)
2 (Moderately Differentiated)	14 (43.75)
3 (Poorly Differentiated)	14 (43.75)
Estrogen Receptor (ER)/Progesterone Receptor (PR) Expression (N, %)	
ER-Positive/PR-Positive	20 (62.5)
ER-Positive/PR-Negative	3 (9.4)
ER-Negative/PR-Negative	9 (28.1)
HER2 Expression (N, %)	
(0–1+) Negative	25 (78.1)
(2+) Equivocal by IHC/Negative with FISH	7 (21.9)

IHC, immunohistochemistry; FISH, fluorescence in situ hybridization.

## Data Availability

Data will be provided upon reasonable request.
